# *IL17A *genetic variation is associated with altered susceptibility to Gram-positive infection and mortality of severe sepsis

**DOI:** 10.1186/cc10515

**Published:** 2011-10-25

**Authors:** Taka-aki Nakada, James A Russell, John H Boyd, Keith R Walley

**Affiliations:** 1 University of British Columbia, Critical Care Research Laboratories, Heart + Lung Institute, St. Paul's Hospital, 1081 Burrard Street, Vancouver, BC, V6Z 1Y6, Canada

## Abstract

**Introduction:**

Interleukin 17A (*IL17A*) plays a key role in host defense against microbial infection including Gram-positive bacteria. Genetic factors contribute to the host defense, but the role of *IL17A *single nucleotide polymorphisms (SNPs) has not yet been investigated in severe sepsis. Therefore, we hypothesized that SNPs in the *IL17A *gene alter susceptibility to infection and clinical outcome of severe sepsis.

**Methods:**

We tested for the association of *IL17A *SNPs with susceptibility to infection and clinical outcome of severe sepsis using two cohorts of European ancestry (derivation cohort, St Paul's Hospital (SPH), *n *= 679; validation cohort, Vasopressin and Septic Shock Trial (VASST), *n *= 517). The primary outcome variable was susceptibility to Gram-positive bacterial infection. The secondary outcome variable was 28-day mortality.

**Results:**

Of four tested *IL17A *tag SNPs (rs4711998, rs8193036, rs2275913, rs1974226), rs1974226 SNP was associated with altered susceptibility to Gram-positive infection in the derivation SPH cohort (corrected *P *= 0.014). Patients having the rs1974226 GG genotype were more susceptible to Gram-positive infection, compared to AG/AA genotype in the two cohorts of severe sepsis (SPH, *P *= 0.0036, odds ratio (OR) 2.19, 95% confidence interval (CI) 1.28-3.72; VASST, *P *= 0.011, OR 1.95, 95%CI 1.16-3.27) and in the subgroup having lung infection (*P *= 0.017, OR 1.90, 95%CI 1.12-3.21). Furthermore, the *IL17A *rs1974226 G allele was associated with increased 28-day mortality in two cohorts (SPH, adjusted OR 1.44, 95%CI 1.04-2.02, *P *= 0.029; VASST, adjusted OR 1.67, 95%CI 1.17-2.40, *P *= 0.0052).

**Conclusions:**

*IL17A *genetic variation is associated with altered susceptibility to Gram-positive infection and 28-day mortality of severe sepsis.

## Introduction

Interleukin 17A (*IL17A*) plays a key role in host defense against infection and development of inflammatory diseases [1-5]. IL17A production is increased as an innate response to bacterial infection in human immune cells [6,7], and elevated serum IL17A levels are observed in human [8] and animal models of sepsis [9,10]. In contrast, deficiency of the IL17 response impairs bacterial clearance, delays recovery from infection [1,11] and increases susceptibility to infection [12-15].

Recently two autosomal mutations of IL17 pathway genes were identified in patients with chronic mucocutaneous candidiasis disease, which is characterized by recurrent or persistent mucocutaneous infections by *Candida albicans *and *Staphylococcus aureus *[16]. The mutations, single nucleotide substitutions in the coding region of *IL17 *genes, abolish the IL17 immune response leading to persistent infections [16]. IL17 deficiency appears to differentially increase susceptibility to infection including *S. aureus *[12] and *C. albicans *[13-15]. These discoveries highlight the importance of the *IL17A *gene on infection and, in particular, on Gram-positive and fungal infections. Severe sepsis is a leading cause of death in intensive care units (ICUs) [17]. Multiple studies have shown that single nucleotide polymorphisms (SNPs) in important immune response genes alter susceptibility to infection and/or outcome of severe sepsis [18-21], but the role of *IL17A *SNPs has not yet been investigated in severe sepsis. Gram-positive infections, including *S. aureus*, are very common pathogens isolated from severe sepsis patients [22,23], while fungal infections are somewhat less common. Thus, we tested the hypothesis that *IL17A *genetic polymorphisms alter susceptibility of patients to Gram-positive infection using isolated pathogens from patients in two large severe sepsis cohorts of European ancestry. We subsequently tested for association of *IL17A *genotype with survival from severe sepsis.

## Materials and methods

### Patients

#### St Paul's Hospital (SPH) Cohort

Severe sepsis was defined by the presence of two or more diagnostic criteria for the systemic inflammatory response syndrome [24], proven or suspected infection, and at least one new organ dysfunction by Brussels criteria [25]. Septic shock was defined by severe sepsis plus hypotension [25] despite adequate fluid resuscitation. All patients admitted to the ICU at St Paul's Hospital (SPH) in Vancouver, Canada between July 2000 and January 2004 were screened (*n *= 1, 626). Of these, 691 patients of European ancestry had severe sepsis, were extensively phenotyped [26], and had DNA available. Twelve patients, who were also enrolled in the Vasopressin and Septic Shock Trial (VASST) [25] were excluded from this cohort to avoid duplication. Thus, 679 severe sepsis patients, including 453 septic shock patients, were analyzed in this study. The Institutional Review Board at SPH and the University of British Columbia (UBC) approved the study. For this fully anonymized analysis the UBC and SPH Review Boards waived the need for informed patient consent.

#### Vasopressin and Septic Shock Trial (VASST) Cohort

VASST was a multicenter, randomized, double blind, and controlled trial evaluating the efficacy of vasopressin versus norepinephrine in a total of 778 septic shock patients [25]. Of these, 517 patients of European ancestry had DNA available and were included in the analysis. The research ethics boards of all participating institutions approved this trial and written informed consent was obtained from all patients or their authorized representatives. The research ethics board at the coordinating center (UBC) approved the genetic analysis.

### Microbiology

We assessed all microbiological cultures taken at the time of admission to the ICU for patients in the SPH cohort. Results from cultures that were collected from 48 hours prior to enrollment to 48 hours after enrollment in the VASST cohort [25] were analyzed. We defined bacterial infection in this study as clinical evidence of infection associated with positive microbiological culture, excluding the cultures judged to be positive due to contamination or colonization by the attending physician. We categorized positive microbial cultures into Gram-positive bacteria, Gram-negative bacteria or fungus since these are the three major pathogen categories indentified in previous studies of sepsis in ICUs [23,27], and included in the analysis. The source of a positive culture was categorized as lung (sputum), blood, abdomen (peritoneal fluid, abscess drainage, biliary tract), skin (soft tissues or wounds), genitourinary system (urine) or other.

### Selection of SNPs and genotyping

Tag SNPs for *IL17A *gene were identified using a multi-marker tagging algorithm of Tagger software [28]. To identify tag SNPs, we used the sequence of the *IL17A *gene plus 2, 000 bp of 5' upstream sequence and considered all SNPs with a minor-allele frequency (MAF) > 10% in the HapMap Phase 2+3 Utah residents with Northern and Western European ancestry from the Centre d'Etude du Polymorphisme Humain (CEPH) collection (CEU) data [29] composed of 174 individuals and chose an r^2 ^threshold of 0.5. This identified four tag SNPs (rs4711998, rs8193036, rs2275913, rs1974226), which were genotyped in the SPH derivation cohort. *IL17A *rs1974226 was genotyped in the VASST replication cohort. DNA was extracted from the buffy coat of discarded blood samples using a QIAamp DNA maxi kit (Qiagen, Mississauga, ON, Canada) and genotyped using the Illumina Golden Gate assay (Illumina, San Diego, CA). We performed quality control of the genotyping using 5% repeat and HapMap Coriell DNA controls. For 5% repeat genotyping and Coriell controls, the concordance rate was > 99%. We did not discard any of the *IL17A *SNPs based on the quality control metrics.

### Statistical analysis

The primary outcome variable was susceptibility to Gram-positive infection. To screen the four SNPs of the *IL17A *gene in the derivation cohort we used an Armitage's trend test followed by Bonferroni correction for multiple comparisons. Power for the genetic association of susceptibility to infection by Gram-positive bacteria was calculated using the Genetic Power Calculator [30]. We tested a joint analysis across the SPH and VASST cohorts on genetic susceptibility to Gram-positive, Gram-negative infection and Gram-positive infection by site of infection using a logistic regression controlling for the cohort. For the secondary analysis, we chose logistic regression to test for the genetic effect on 28-day mortality to allow for correction of potential confounding factors, including age, gender, surgical versus medical primary diagnosis and septic shock as covariates.

We tested for differences in baseline characteristics by genotype using a chi-square test for categorical data and a Kruskal Wallis test for continuous data. We tested for Hardy-Weinberg equilibrium using a chi-square test (threshold *P*-value < 0.05 was considered significant). We tested for differences in survival curves using a log-rank test for trend. R2 measure of linkage disequilibrium was calculated using HapMap Phase 3 genotyping data (Han Chinese in Beijing (HCB), Japanese in Tokyo (JPT), CEU) in the genome variation server [31]. Differences were considered significant using a two-tailed *P *< 0.05. Analyses were performed using R (version 2.8.1) [32] and SPSS (SPSS, version 16, Chicago, IL) statistical software packages.

## Results

A total of 679 severe sepsis patients of European ancestry in the derivation SPH cohort were successfully genotyped for four tag SNPs of *IL17A*. Patients with a positive microbiological culture (*n *= 301) had similar baseline characteristics to the entire population in the derivation cohort (Table 1) and had similar allele frequencies to those of HapMap European ancestry data (Table 2). *IL17A *is reported to be essential for host defense against Gram-positive bacterial infection [1]. We first tested for the association of four SNPs with susceptibility to Gram-positive infection using the Armitage trend test for the additive model in the derivation cohort (Table 2). Of the four SNPs, the major G allele of *IL17A *rs1974226 G/A was significantly associated with increased susceptibility to infection by Gram-positive bacteria (percentages of positive culture: AA 38.5%, AG 46.4%, GG 64.2%, uncorrected *P *= 0.0037, Bonferroni corrected *P *= 0.014) (Table 2).

**Table 1:** Characteristics of culture-positive patients in two cohorts of severe sepsis

	SPH		VASST	
	Culture Positive	All	Culture Positive	All
	(*n *= 301)	(*n *= 679)	(*n *= 282)	(*n *= 517)
Age-years	58 (45-71)	59 (46-72)	63 (50-73)	63 (51-73)
Gender -% male	65.4	66.0	60.3	60.9
APACHE II	24 (18-30)	23 (18-29)	26 (21-31)	26 (21-32)
Surgical -%	26.6	28.7	19.1	21.3
Septic shock-number (%)	208 (69.1)	453 (66.7)	282 (100)	517 (100)
Pathogens-number (%)				
Gram-positive bacteria alone	152 (50.5)		115 (40.8)	
Gram-negative bacteria alone	100 (33.2)		82 (29.1)	
Fungus alone	9 (3.0)		37 (13.1)	
Mixed	40 (13.3)		48 (17.0)	
Source of a positive culture-number (%)				
Lung	126 (41.9)		140 (49.6)	
Blood	115 (38.2)		131 (46.5)	
Abdomen	20 (6.6)		53 (18.8)	
Skin and soft tissue	21 (7.0)		36 (12.8)	
Genitourinary system	16 (5.3)		17 (6.0)	
Other	3 (1.0)		29 (10.3)	

APACHE, Acute Physiology and Chronic Health Evaluation; SPH, St. Paul's Hospital; VASST, Vasopressin and Septic Shock Trial

Data are median (interquartile range) for continuous variables.

**Table 2:** Allele frequency and association of *interleukin17A *polymorphisms with Gram-positive bacterial infection in the derivation cohort of severe sepsis.

	Location^a^ base pair	Major/minor allele	MAF (HapMap^b^)	HWE *P *value^c^	Odds Ratio (95% CI)	*P *value^d^ (Corrected *P *value^e^)
rs4711998	-832	G/A	0.326 (0.217)	0.025	1.13 (0.80-1.60)	0.48
rs8193036	-692	T/C	0.287 (0.241)	0.53	1.41 (0.97-2.05)	0.073
rs2275913	-399	G/A	0.331 (0.383)	0.79	1.45 (1.01-2.10)	0.045 (0.18)
rs1974226	+4150 (3' UTR)	G/A	0.182 (0.179)	0.10	1.89 (1.22-2.92)	0.0037 (0.015)

HWE, Hardy-Weinberg equilibrium; MAF, minor allele frequency; UTR, untranslated region.

^a^Relative distance to transcription start site; ^b^Minor allele frequency of HapMap Utah residents with Northern and Western European ancestry from the Centre d'Etude du Polymorphisme Humain collection (CEU) data; ^c^Hardy-Weinberg equilibrium *P *values for culture positive patients were calculated using the chi-square test; ^d^*P *values were calculated using Armitage's test and corrected for multiple comparison by a Bonferroni test^e^.

**Figure 1:**
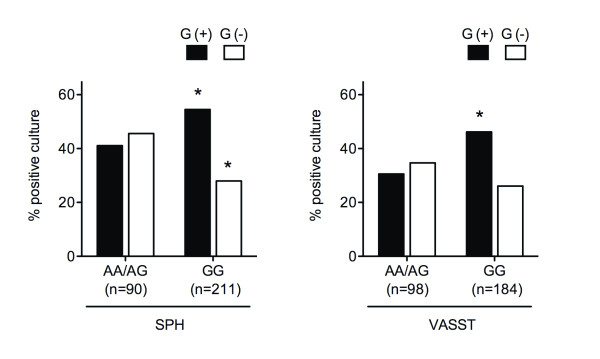
**Frequency of patients who had Gram-positive or Gram-negative bacterial culture-positive by *IL17A *rs1974226 genotype in two cohorts of severe sepsis**. *IL17A *rs1974226 GG genotype patients had an increased culture-positive rate of Gram-positive bacteria compared to the AA/AG genotype patients and a decreased rate of Gram-negative bacteria in two cohorts (AA/AG versus GG, SPH, Gram-positive *P *= 0.0036, Gram-negative *P *= 0.0086; VASST, Gram-positive *P *= 0.011, Gram-negative *P *= 0.13). SPH, St Paul's Hospital; VASST, Vasopressin and Septic Shock Trial. *P *values were calculated using chi-square test. *P < 0.05

Whether the observation that *IL17A *rs1974226 genotype altered susceptibility to infection, is specific to Gram-positive bacteria is unknown. Therefore, we next tested for genetic association of altered susceptibility to infection by three pathogen categories including Gram-positive bacteria, Gram-negative bacteria and fungus in the SPH derivation cohort. Due to the small sample size of the minor homozygote AA genotype patients who had a positive culture (SPH AA genotype; Gram-positive *n *= 5, Gram-negative *n *= 8, fungus *n *= 0, mixed *n *= 1, total *n *= 14), we compared AA+AG genotype versus GG genotype in this analysis. In the derivation SPH cohort, the GG genotype patients had increased susceptibility to Gram-positive bacterial infection (AA+AG vs. GG, Gram-positive, *P *= 0.0036, odds ratio (OR) 2.19, 95% confidence interval (CI) 1.28-3.72) and decreased susceptibility to Gram-negative bacterial infection (AA+AG vs. GG, Gram-negative, *P *= 0.0086, OR 0.49 95%CI 0.29-0.84) (Figure 1). We subsequently tested for replication using the validation VASST cohort of European ancestry, which was successfully genotyped for *IL17A *rs1974226. VASST patients with a positive microbiological culture (*n *= 282) had a similar allele frequency (MAF = 0.195) compared to HapMap and SPH (Table 1) and were in Hardy-Weinberg equilibrium (*P *= 0.63). In accord with the observation in the derivation cohort, patients having the GG genotype had increased susceptibility to Gram-positive bacterial infection compared to those with the AA+AG genotype in the replication cohort (*P *= 0.011, OR 1.95, 95%CI 1.16-3.27) (Figure 1). When we calculated power to detect an association between *IL17A *rs1974226 genotype (GG versus AG/AA) and Gram-positive infection, we used the observed prevalences (Gram-positive bacteria, 0.505 (SPH), 0.408 (VASST)), observed allele frequencies and a relative risk = 2.0. We found that our study had a 99.8% power for SPH and 98.0% power for VASST (alpha = 0.05) of detecting a genotype effect. Joint analysis across the SPH and VASST cohorts using a logistic regression controlling for the cohorts yields the same conclusion for Gram-positive bacterial infection (AA+AG versus GG, *P *= 1.4 × 10^-4^, OR 2.06, 95%CI 1.42-2.99). A non-significant trend in the same direction of altered susceptibility to Gram-negative infection was observed in the VASST cohort (*P *= 0.13, OR 0.66, 95%CI 0.39-1.13). In the joint analysis across the SPH and VASST cohorts, the GG genotype patients had decreased susceptibility to Gram-negative bacterial infection (AA+AG versus GG, *P *= 0.0035, OR 0.57, 95%CI 0.39-0.83). There was no difference of genetic susceptibility to fungal infection in the two cohorts (AA/AG versus. GG, SPH, 4.9% versus 2.8%, *P *= 0.33; VASST, 15.3% versus 12.0%, *P *= 0.43), however the number of patients having a fungal infection was low so that this negative result has limited statistical power.

**Figure 2:**
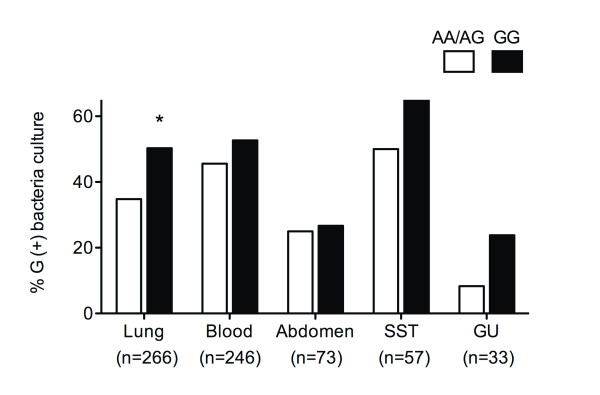
**Frequency of Gram-positive bacteria infection by site**. Patients who had the GG genotype of *IL17A *rs1974226 SNP had significantly increased Gram-positive infection compared to AA/AG genotype in the lung, and non-significant trends in the same direction were observed in other sites (AA/AG versus GG, SPH+VASST, lung, *P *= 0.017; blood, *P *= 0.53; abdomen, *P *= 0.91; skin and soft tissue [SST], *P *= 0.22; genitourinary system [GU], *P *= 0.25). SPH, St Paul's Hospital; VASST, Vasopressin and Septic Shock Trial. *P *values were calculated using a logistic regression controlling for the cohort. *P < 0.05

We further tested for the altered genetic susceptibility to Gram-positive bacteria by site of infection (Table 1). We combined two cohorts due to smaller subset sample size and analyzed using a logistic regression controlling for the cohort. The GG genotype patients had significantly increased Gram-positive bacterial infections compared to the AA/AG genotype in the lung (*P *= 0.017, OR 1.90, 95%CI 1.12-3.21), and non-significant trends in the same direction were observed in the other sites (blood, *P *= 0.53; abdomen, *P *= 0.91; skin and soft tissue (SST), *P *= 0.22; genitourinary system (GU), *P *= 0.25) (Figure 2).

**Figure 3:**
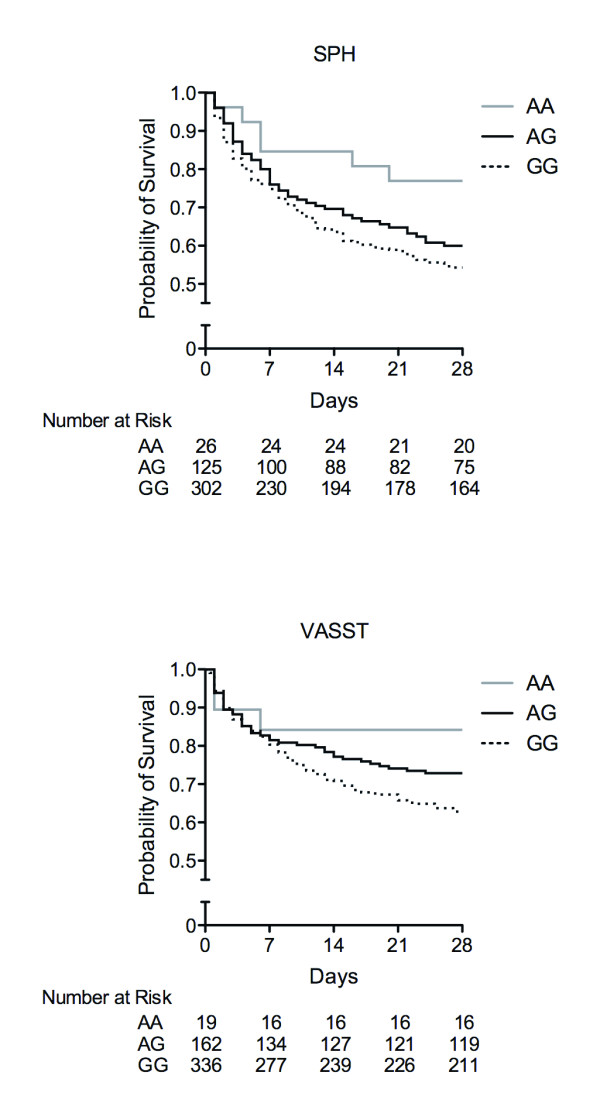
**Survival curves over 28 days by *IL17A *rs1974226 genotype in two cohorts of septic shock**. Patients with IL17A rs1974226 GG genotype had increased mortality in the SPH and VASST cohort of septic shock compared to the AG or AA genotype patients (SPH *P *= 0.029, VASST, *P *= 0.010). SPH, St Paul's Hospital; VASST, Vasopressin and Septic Shock Trial. *P *values were calculated using a log-rank test for trend.

We next tested whether the *IL17A *rs1974226 altered the 28-day mortality of septic shock in the two cohorts. Because VASST only included septic shock patients, we limited our analysis of the SPH cohort to septic shock in order to examine cohorts of similar overall severity of illness. There was no baseline difference by the genotype of rs1974226 in age, gender, Acute Physiology and Chronic Health Evaluation (APACHE) II, surgical versus medical, pre-existing conditions, physiological or laboratory variables in the SPH cohort (Table 3). In the VASST cohort, patients who had rs1974226 GG or AG genotype had a decreased Pa_O2_/F_IO2 _ratio compared to AA genotype in parallel with an increased rate of GG or AG genotype patients of chronic lung disease (Table 3). The GG genotype patients had increased mortality over 28 days compared to the AG or AA genotype in the two cohorts of septic shock (Figure 3, SPH, *P *= 0.029; VASST, *P *= 0.010, log-rank test for trend: Table 4, G allele; SPH (septic shock), adjusted OR 1.44, 95%CI 1.04-2.02, *P *= 0.029; VASST, adjusted OR 1.67, 95% CI 1.17-2.40, *P *= 0.0052, logistic regression). In addition, repeating the analysis of the SPH cohort including all patients in a logistic regression analysis adjusted by including septic shock as a covariate yielded the same conclusions (SPH (severe sepsis), adjusted OR 1.35, 95% CI 1.01-1.80 *P *= 0.042).

**Table 3:** Baseline characteristics in two cohorts of severe sepsis patients by the genotype of *IL17A *rs1974226 polymorphism.

	SPH (*n *= 679)				VASST (*n *= 517)			
*IL17A *rs1974226 G/A	AA	AG	GG		AA	AG	GG	
	(*n *= 39)	(*n *= 174)	(*n *= 466)	*P *	(*n *= 19)	(*n *= 162)	(*n *= 336)	*P *
Age-years	60(47-73)	60(47-71)	59(46-72)	0.96	60(47-71)	66(51-74)	63(51-72)	0.57
Gender -% male	76.9	60.3	67.2	0.089	57.9	55.6	63.7	0.21
APACHE II	21(17-28)	23(17-29)	23(18-29)	0.74	25(19-35)	26(21-32)	27(22-31)	0.63
Surgical -%	30.8	32.2	27.3	0.45	21.1	22.8	20.5	0.84
Septic shock-n(%)	26 (5.7)	125 (27.6)	302 (66.7)	0.24	19 (3.7)	162 (31.3)	336 (65.0)	NA
Preexisting disease-n(%)								
Chronic heart failure	2(5.1)	13(7.5)	25(5.4)	0.59	1(5.3)	13(8.0)	27(8.0)	0.91
Chronic lung disease	5(12.8)	41(23.6)	79(17.0)	0.10	1(5.3)	39(24.1)	55(16.4)	0.037
Chronic liver disease	4(10.3)	16(9.2)	49(10.5)	0.89	1(5.3)	15(9.3)	36(10.7)	0.69
Chronic renal failure	1(2.6)	9(5.2)	15(3.2)	0.47	2(10.5)	16(9.9)	36(10.7)	0.96
Vasopressin-n(%)	3(7.7)	27(15.5)	63(13.5)	0.43	9(47.4)	86(53.1)	168(50.0)	0.77
Corticosteroids^a ^-n(%)	9(23.1)	68(39.1)	156(33.5)	0.13	11(57.9)	85(52.5)	184(54.8)	0.84
Corticosteroids-day	4(1-20)	6(2-16)	4(2-12)	0.61	10(6-21)	8(5-16)	8(4-14)	0.64
Activated protein C-n(%)	1(2.6)	4(2.3)	22(4.7)	0.34	0(0)	27(16.7)	43(12.8)	0.11
Variables-Day1								
Body temperature-C°	37.8(37.1-38.5)	37.9(37.0-38.6)	37.9(36.6-38.6)	0.73	38.8(37.6-39.3)	38.5(37.5-39.1)	38.5(37.6-39.3)	0.85
Heart rate -/min	115(105-120)	106(95-126)	112(95-130)	0.67	126(107-140)	128(115-140)	125(106-137)	0.12
MAP-mmHg	59(52-71)	57(51-65)	58(52-68)	0.34	58(51-63)	56(50-61)	55(50-61)	0.60
WBC-10^3^/mm^3^	12.9(9.0-17.1)	15.0(9.3-21.7)	13.5(9.3-18.9)	0.075	14.3(4.6-19.6)	13.4(8.3-19.9)	13.9(7.7-21.3)	0.69
Platelet-10^3^/mm^3^	149(97-183)	191(113-261)	171(95-259)	0.091	150(123-224)	145(74-246)	165(86-263)	0.44
Pa_O2_/F_IO2 _ratio-mmHg	162(106-234)	154(106-224)	158(98-229)	0.84	233(182-317)	198(153-263)	190(131-251)	0.040
Creatinine -μmol/L	165(73-295)	112(73-240)	126(80-231)	0.50	190(96-297)	149(90-228)	154(90-270)	0.38

APACHE, Acute Physiology and Chronic Health Evaluation; MAP, mean arterial pressure; n, number; NA, not applicable; SPH, St Paul's Hospital; VASST, Vasopressin and Septic Shock Trial; WBC, white blood cell count.

^a^Corticosteroids, low dose corticosteroid.

Data are median (interquartile range) for continuous variables. *P *values were calculated with the use of chi-square test and Kruskal Wallis test.

**Table 4:** Logistic regression analysis of 28-day mortality

	SPH		VASST	
	Odds Ratio (95% CI)	*P*	Odds Ratio (95% CI)	*P*
Age-yearsr	1.029 (1.016-1.042)	1.4 × 10^-5^	1.021 (1.008-1.034)	0.0011
Female	0.99 (0.66-1.49)	0.99	0.92 (0.62-1.35)	0.67
Surgical	0.70 (0.45-1.07)	0.10	0.84 (0.53-1.33)	0.46
*IL17A *rs1974226 G allele	1.44 (1.04-2.02)	0.029	1.67 (1.17-2.40)	0.0052

SPH, St. Paul's Hospital; VASST, Vasopressin and Septic Shock Trial.

Odds ratio was calculated for patients with septic shock using a logistic regression including potential cofounding factors: age, gender (female versus male), surgical versus medical primary diagnosis, and IL17A rs1974226 G allele (GG versus GA versus AA) as covariates.

## Discussion

We found that patients who had the GG genotype of *IL17A *rs1974226 G/A SNP had increased susceptibility to Gram-positive bacterial infection in the derivation cohort and this result was replicated in the validation cohort. In addition, this genotype was significantly associated with adverse clinical outcome of severe sepsis. We found that the G allele of the *IL17A *SNP was associated with increased 28-day mortality in both cohorts of severe sepsis/septic shock patients.

IL17A is an essential cytokine for host defense against bacteria [1,9], which is produced by a variety of cells [2,3] including T cells (T helper 17, γδ T, NKT cell), lymphoid-tissue inducer-like cells [33], neutrophils [34] and intestinal Paneth cell [35]. Cells triggered by microbes secrete IL17A, which is then recognized by an IL17 receptor [2-4]. Subsequent cellular signaling activates inflammatory pathways including NF-kB and MAPK/AP-1, which invoke production of pro-inflammatory cytokines, chemokines and antimicrobial peptides, which induce inflammation required for host defense [2-4]. Human IL17A-producing cells increase IL17A production against bacteria or their toxins [6,7]. Serum IL17A levels were elevated in bacterial sepsis patients with acute respiratory distress syndrome (ARDS) [8] and in animal models of abdominal bacterial infection [9,10]. IL17 deficiency in mice impairs microbial clearance, delays recovery from infection [1,11] and increases susceptibility to infection including *S. aureus *[12] and *C. albicans *[13-15]. In accord with the observations in IL17 deficient mice, low birth weight infants who had blood stream infections had deceased blood IL17 levels compared to those without blood stream infections [36]. These results are in accord with our current observations that severe sepsis patients who had the *IL17A *rs1974226 GG genotype had increased susceptibility to Gram-positive infection.

The human *IL17A *gene is composed of three exons (two introns) and located on chromosome 6p12 covering 4, 252 bases of genomic DNA. The transcript (1, 859 bp) has a relatively long 3'UTR region (1, 345 bp), where the rs1974226 SNP is located. Since the 3'UTR is involved in regulation of gene expression, such as mRNA stability and/or degradation as well as translation efficiency [37,38], a potential mechanism of the rs1974226 effect is alteration of gene regulation. Alternatively, other SNPs in high linkage disequilibrium with this SNP may have a biological function. In the Caucasian population the at-risk allele (G) is the major allele of rs1974226 G/A, potentially marking a haplotype that includes many rare functional SNPs that increase risk, or the rarer A allele may actually be protective. Of the four *IL17A *SNPs screened in this study, two *IL17A *SNPs (rs8193036 and rs2275913) were associated with susceptibility to inflammatory diseases, such as pediatric asthma (rs8193036, risk CC genotype, Taiwan) [39], ulcerative colitis (rs2275913, risk A allele, Japan) [40] and rheumatoid arthritis (rs2275913, risk GG allele, Norway and New Zealand) [41]. These reported SNPs are not in high linkage disequilibrium with rs1974226 (rs8193036 r^2 ^= 0.028 (HapMap HCB), rs2275913, r^2 ^= 0.028 (HapMap JPT), r^2 ^= 0.135 (HapMap CEU)). Thus, our finding regarding rs1974226 is not identical to these previous reports but similarly leads to the conclusion that genetic variation in the *IL17A *gene alters outcome from a variety of inflammatory disorders.

The GG genotype was associated with increased susceptibility to Gram-positive bacterial infection in two cohorts of severe sepsis patients, whereas a trend towards decreased susceptibility to Gram-negative bacteria was also observed. Genetic associations of pathogen specific susceptibility have been documented in pattern recognition receptors (PRRs) such as Toll-like receptors (TLRs) [42]. The potential mechanism of the different pathogen-specific susceptibility may be difference of PRRs.

IL17 induces neutrophil recruitment in the airways [43], which is an important mechanism of host defense for the lung. In accordance with this, we found that the rs1974226 GG genotype patients had significantly increased susceptibility to Gram-positive infection in the lung. While this association was significant for the lung, associations were not significant for other sites. This might be due to the small sample sizes of infections found in other sites. Other limitations of this study are, first, that we did not investigate mechanisms of action such as altered *IL17A *gene expression or alternative splicing by the *IL17A *rs1974226 SNP. Thus, further investigation regarding the genetic effect on IL17A mRNA structure and mRNA expression levels and protein levels would strengthen the results of this study. Second, the GG genotype had increased susceptibility to Gram-positive infection and 28-day mortality, but the causal link is not proven in this study. Third, this study finding of an association of the *IL17A *rs174226 GG genotype with increased susceptibility to a category of Gram-positive bacteria would be strengthened by testing for an association with a specific Gram-positive bacterial pathogen, such as *S. aureus*, in further larger studies.

Decreased susceptibility to Gram-negative bacteria observed in the GG genotype patients in the derivation cohort did not replicate; this needs further replication tests in larger cohorts.

## Conclusions

To conclude, the *IL17A *rs1974226 GG genotype is associated with increased numbers of Gram-positive infections and increased 28-day mortality in severe sepsis patients.

## Key messages

• Patients of European ancestry having the *IL17A *rs1974226 GG genotype were more susceptible to Gram-positive infection, compared to those having the AG/AA genotype in the derivation and validation cohorts of severe sepsis.

• The *IL17A *rs1974226 GG genotype patients had significantly increased Gram-positive bacterial infection compared to the AA/AG genotype in the subgroup having lung infection.

• The *IL17A *rs1974226 G allele was associated with increased 28-day mortality in both cohorts of severe sepsis/septic shock patients.

## Abbreviations

APACHE: Acute Physiology and Chronic Health Evaluation; ARDS: Acute respiratory distress syndrome; GU: genitourinary system; HWE: Hardy-Weinberg equilibrium; IL17A: Interleukin 17A; MAF: Minor-allele frequency; SST: Skin and soft tissue; SNPs: Single nucleotide polymorphisms; SPH: St Paul's Hospital; UTR: Untranslated region; VASST: Vasopressin and Septic Shock Trial.

## Competing interests

TN and JHB have no potential conflicts of interest to disclose. JAR and KRW hold shares in Sirius Genomics Inc., which has submitted patents owned by UBC and licensed to Sirius Genomics Inc., that are related to the genetics of vasopressin and protein C. JAR has received consulting fees from Ferring, which manufactures vasopressin, from Astra Zeneca which manufactures anti-TNF, and from Sirius Genomics Inc, and has received grant support from Sirius Genomics, Novartis, Ferring, and Eli Lilly, and has received speaking honoraria from Pfizer and Eli Lilly.

## Authors' contributions

TN and KRW contributed to study conception and design, acquisition of data, statistical analysis, interpretation of data, and drafting of the manuscript. JAR and JHB contributed to study conception and design, acquisition of data, interpretation of data, and drafting of the manuscript. All the authors read and approved the final manuscript.
